# Hypo-fractionated stereotactic radiotherapy alone using volumetric modulated arc therapy for patients with single, large brain metastases unsuitable for surgical resection

**DOI:** 10.1186/s13014-016-0653-3

**Published:** 2016-06-02

**Authors:** Pierina Navarria, Federico Pessina, Luca Cozzi, Anna Maria Ascolese, Fiorenza De Rose, Antonella Fogliata, Ciro Franzese, Davide Franceschini, Angelo Tozzi, Giuseppe D’Agostino, Tiziana Comito, Cristina Iftode, Giulia Maggi, Giacomo Reggiori, Lorenzo Bello, Marta Scorsetti

**Affiliations:** Radiosurgery and Radiotherapy Department, Istituto Clinico Humanitas Cancer Center and Research Hospital, Rozzano Milan, Italy; Neuro Surgery Department, Istituto Clinico Humanitas Cancer Center and Research Hospital, Rozzano Milan, Italy

**Keywords:** HSRT, Volumetric modulated arc therapy, RapidArc, Brain metastases

## Abstract

**Background:**

Hypo-fractionated stereotactic radiotherapy (HSRT) is emerging as a valid treatment option for patients with single, large brain metastases (BMs). We analyzed a set of our patients treated with HSRT. The aim of this study was to evaluate local control (LC), brain distant progression (BDP), toxicity and overall survival (OS).

**Methods:**

From July 2011 to May 2015, 102 patients underwent HSRT consisting of 27Gy/3fractions for lesions 2.1–3 cm and 32Gy/4 fractions for lesions 3.1–5 cm. Local progression was defined as increase of the enhancing abnormality on MRI, and distant progression as new brain metastases outside the irradiated volume. Toxicity in terms of radio-necrosis was assessed using contrast enhanced T1MRI, T2 weighted-MRI and perfusion- MRI.

**Result:**

The median maximum diameter of BM was 2.9 cm (range 2.1–5 cm), the median gross target volume (GTV) was 16.3 cm^3^ and the median planning target volume (PTV) was 33.7 cm^3^ The median,1,2-year local control rate was 30 months, 96, 96 %; the median, 1–2-year rate of BDP was 24 months, 12, 24 %; the median,1,2-year OS was 14 months, 69, 33 %. KPS and controlled extracranial disease were associated with significant survival benefit (*p* <0.01). Brain radio-necrosis occurred in six patients (5.8 %).

**Conclusion:**

In patients with single, large BMs unsuitable for surgical resection, HSRT is a safe and feasible treatment, with good brain local control and limited toxicity.

## Background

Brain metastases (BMs) occur in 20–40 % of adult cancer patients and the incidence increased two to five times over the last 40 years [1, 2]. In cases of single, large BMs, the treatment approach includes surgical resection, whole brain radiation therapy (WBRT) and stereotactic radiosurgery (SRS). Although surgical resection is the main treatment option, many patients are unsuitable for surgery due to their general condition, Karnofsky Performance Scale (KPS), age and comorbidity, critical location of lesions or uncontrolled primary tumor and/or extracranial metastatic site. For several years, WBRT has been considered the standard of care for these cancer patients but considering the poor local control (LC) rate in the case of large brain lesions [3], other radiation therapy modalities were investigated. SRS, whether combined or not with WBRT, is increasingly used for patients with solitary or limited BMs (up to four) with a recorded local control of 70–90 % at 12 months [4–7]. However, SRS, using the dose guidelines recommended by the Radiation Therapy Oncology Group (RTOG) 90-05 study, achieves a LC of 49 % in metastases between 2.1 and 3 cm and of 45 % in metastases between 3.1 and 4.0 cm [7]. On this topic, literature data showed a 3-fold increased risk of local failure for tumor treated with 15–18Gy compared to 24Gy, with a 1-year local control <50 % [8]. On the other hand, single large doses may be associated with an increased risk of neurologic morbidity from radiation necrosis, and this is of concern especially for lesions larger than 2.5–3.0 cm or that are in close proximity to critical structures, such as optic apparatus or brainstem [8–13]. In such cases, hypo-fractionated stereotactic radiotherapy (HSRT) using up to five fractions was employed with the aim to maintain a high local control rate whilst decreasing the late radiation-induced toxicity. There is limited evidence for the treatment of larger brain metastases, specifically those greater than 3 cm in maximum diameter. Many of published studies, included patients treated both for small and for large lesions and results were not stratified according to the tumor size [14–25]. In our department, we choose to treat patients with single large (≥2.1 cm) BMs, unsuitable for surgical resection, using a multi-fraction stereotactic radiotherapy rather than SRS in a single session. This analysis concerns only patients with single large BMs treated in this way. Primary objective was to evaluate the safety and the feasibility of HSRT in terms of toxicity and its impact on brain LC. In addition, brain distant progression (BDP) and overall survival (OS) were evaluated.

## Methods and materials

### Patients and procedures

**Fig. 1:**
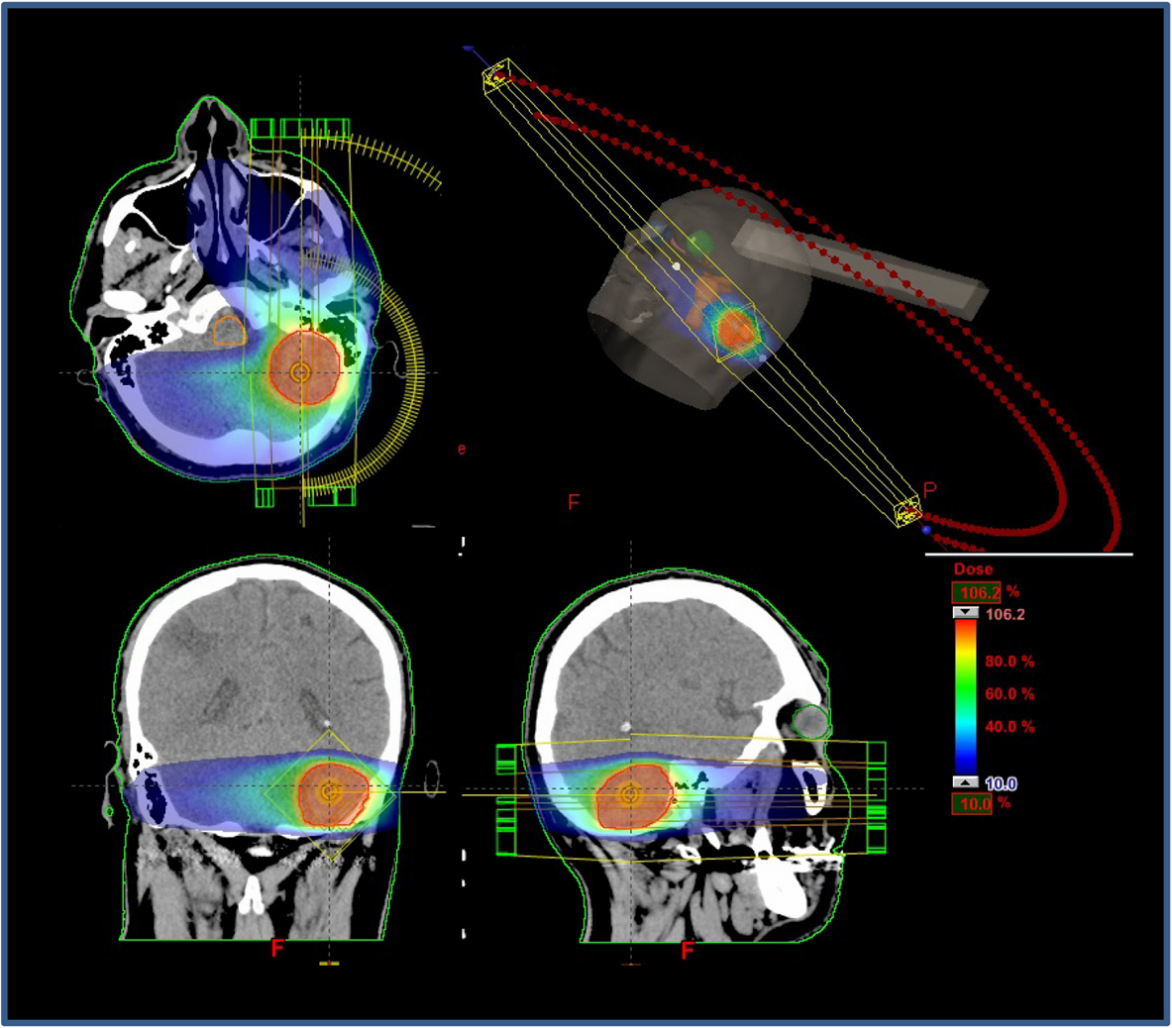
Treatment plan of patient with left cerebellum metastases by Volumetric modulated arc therapy (VMAT) using two partial co-planar arcs

The present retrospective study includes patients with single large BMs. All patients were treated in agreement with the Helsinki declaration. This study was based on a retrospective analysis of treatment charts and received approval by the Humanitas Hospital Ethical Committee. All patients, during admission, signed a consent to the use of their data for scientific scopes. To define the appropriate therapy, each patient was evaluated by a multidisciplinary team including a neurosurgeon, a neuro-oncologist and a radiation oncologist. HSRT was performed when at least one of the following conditions was met: 1) KPS ≥70, 2) oligo-metastatic disease, defined as the presence of up to a total of five metastatic lesions, both cranial and extracranial, 3) histological diagnosis of primary solid tumor excluding small cell lung cancer (SCLC) and lympho-proliferative disease, 4) single, large BMs (≥2.1 cm), 5) contra-indication to surgical resection for patients general condition, uncontrolled extra-cranial metastases, and critical location of BMs. Patients with lesions ≤2 cm were excluded from this analysis, as they undergo single dose stereotactic radiosurgery (SRS). To precisely delineate the target volume, enhanced T1-MRI sequences and post-contrast CT scans were acquired and co-registered. Patients were placed in supine position with arms close to the body. A personalized thermoplastic mask was used for patient immobilization and repositioning. All CT scans extending from the top of the skull to the third cervical vertebrae were acquired with 1 mm slice thickness and imported in the iPlan-net Brainlab stereotactic treatment planning system (Brainlab Ag, Feldkirchen, Germany). An automatic rigid co-registration was performed for all patients. Gross tumor volume (GTV) consisted of the abnormality on enhanced contrast T1-MRI; clinical target volume (CTV) corresponded to GTV and no additional margins were used; planning target volume (PTV) was generated adding an isotropic margin of 3 mm from CTV. This was chosen as a safety upper limit due to the use of relocable thermoplastic masks rather than fixed localizers. Organs at risk (OARs) delineated were brain, brainstem, optic nerves, chiasm and lenses. No margins were added to OARs. All plans were optimized on PTV. The prescribed total dose and fractionation was chosen in relation to the size of BMs and/or to the close proximity of OARs: for lesions between 2.1 and 3 cm the schedule used was 27Gy in three daily fractions, and for lesions between 3.1 and 5 cm or located near to optic apparatus or brain stem was 32Gy in four daily fractions. The objective was to obtain a biological equivalent dose (BED) for tumor (BED_10_) >50Gy. As per the institutional policy in these patients, the planning objective for OARs was to minimize as much as possible the dose to normal brain tissue. The upper dose-volume constraints used for brainstem, optic apparatus, and lenses were D_1%_ ≤20Gy, D_1%_ ≤15Gy, and D_1%_ <1Gy, respectively. The constraint for the mean dose to the brain was 4Gy (i.e. to the entire healthy brain without volume thresholds). No other specific dose-volume constraints were applied to other structures. Plans were optimized aiming to achieve a PTV coverage of D_95%_ >95 % with a homogeneous dose distribution. The AAPM task group 101 suggests prescribing SBRT or SRS at a specific isodose (usually approximately 80 %). However, the modern algorithms of inverse TPS intrinsically intend to obtain homogeneous dose to the PTV, because the systems were primarily designed for conventional fractionated RT treatment. Moreover, ICRU 83 suggests normalizing to a mean value without specifically distinguishing between the standard RT and stereotactic treatments [26]. Normalization was set in order to have 100 % of the dose to target (PTV) mean. All plans were optimized using two partial co-planar or non co-planar arcs, according to the lesion position as shown in Fig. 1. All patients were treated with the volumetric modulated arc technique RapidArc (Varian Medical System, Palo Alto, USA) on three different Varian Linacs equipped with a 4D couch. Exactrac (Brainlab) and Cone Beam CT imaging was performed daily for patient set up and positioning verification.

### Outcome evaluation

Clinical outcome was evaluated by neurological examination and a brain MRI was performed two months after RT and then every 3 months. Local progression was defined as radiographic increase of the enhancing abnormality in the irradiated volume on serial MR imaging. Distant failure, instead, was defined as the presence of new brain metastases or leptomeningeal enhancement outside the irradiated volume. LC was assessed and reported for alive patients while the OS analysis was performed on all patients. Toxicities were graded according to Common Terminology Criteria for Adverse Events version 4.0. Radio-necrosis was assessed using contrast enhanced T1MRI, T2 weighted-MRI and perfusion-MRI. Radio-necrosis was considered as the presence of central hypo-density and peripheral enhancement on T1-weighted post-contrast imaging, with edema on T2-weighted sequences and a clear lack of perfusion without any nodular highly vascularized area within the contrast enhanced lesion on perfusion MRI. Histologic confirmation of radio-necrosis was not required except for patients in which surgical resection has been needed. Patients with uncontrolled extracranial disease, at the first examination time, underwent systemic therapy, chemotherapy, hormonal therapy or biological target therapy, as appropriate for the tumour histology.

### Statistical analysis

Standard descriptive statistics (mean, standard deviation and cross tabulation analysis) were used to describe the general data behavior. Survival and recurrence time observations were plotted according to the method of Kaplan and Meier, starting from the date of HSRT. The log-rank test was used to carry out the univariate analysis, in order to investigate the prognostic role of individual variables. For analysis, variables analyzed were age, KPS, histology of primary tumor, extracranial disease status (controlled/uncontrolled) at the time of HSRT, recursive partitioning analysis (RPA) class, diagnosis- specific Graded Prognostic Assessment (DS-GPA), and size of BMs. Groups were defined according to discrete volume of each variables. For age the analysis was dichotomized according to 65 years threshold. Multivariate Cox model was used as a method to estimate the independent association of a variable set with overall survival (OS), local control (LC), and brain distant progression (BDP). Statistical software used was STATA v. 13.1.

## Results

### Patients and treatments

From July 2011 to May 2015, among patients referred to our institution for BM, 102 patients with single, large BMs eventually underwent HSRT. Of these patients, 39 (38 %) were female and 63 (62 %) male, with a median age of 61 years (range 30–93 years). The most common primary cancers were lung, breast, and melanoma. BMs were present at diagnosis in 51 patients (50 %), whereas they developed in 51 (50 %) after primary tumor treatment. At the time of BMs diagnosis, 66 (65 %) had also additional extracranial metastatic localizations. No patients had uncontrolled primary tumor. Based on RPA class which considers age, KPS, controlled primary tumor and extracranial metastases [27], 18 (18 %) patients were in RPA class I, 81 (79 %) in RPA class II and 3 (3 %) in RPA class III. In relation to GPA score [28] which considers histology of primary tumor, age, KPS, presence of extracranial metastases and number of cranial metastases, 12 (12 %) patients had a GPA score between 0 and 1.0, 54 (53 %) a GPA score between 1.5 and 2.5, 21 (20 %) a GPA score three, and 15 (15 %) a GPA score between 3.5 and 4. Details about tumor specific GPA are shown in Table 1. Fifty-one (50 %) lesions received 27Gy, 9Gy per fraction in three consecutive days and 51 (50 %) lesions received 32Gy, 8Gy per fraction in four consecutive days. Patient’s tumor and treatment characteristics are shown in Table 1. The median maximum diameter of BMs was 2.9 cm (range 2.1–5 cm), the median GTV was 16.3 cm^3^ (range 3.9–64.5 cm^3^) and the median PTV was 33.7 cm^3^ (range 9.2–22.3 cm^3^). Details about GTV and PTV are shown in Table 2. For all patients the required dose objectives for target coverage, organs at risk and for healthy brain were respected and, concerning mean dose to the healthy brain, for the majority of the plans the mean dose was much lower than 4Gy.

**Table 1:** Patients, tumor and treatment characteristics

*Patients*	N. 102				%
*Gender*					
Female	39				38
Male	63				62
Age (median)	61 years (range 30–93 years)				
*Histology*					
Breast Cancer	18				17
NSCLC	57				56
Melanoma	12				12
Other (CCC, Colon)	15				15
*Stage at diagnosis of primary tumor*					
I–III	51				50
IV	51				50
*KPS at BM diagnosis*					
100	18				17
90	18				17
80	63				63
70	3				3
*RPA class*					
I	18				18
II	81				79
III	3				3
*GPA score*	*0–1*	*1.5–2.5*	*3*	*3.5–4*	
Breast cancer	0	6	12	0	
NSCLC	12	36	3	6	
Melanoma	0	0	6	6	
Other	0	12	3	0	
Other extracranial metastatic site at diagnosis of BM	*66*				65
BM median maximum diameter cm (range cm)	2.9 (2.1–5)				
BM diameter cm					
2.1–3 cm	51				50
3.1–5 cm	51				50
GTV median volume cm^3^ (range cm^3^)	16.3 (3.9–64.5)				
PTV median volume cm^3^ (range cm^3^)	33.7 (9.2–122.3)				
HSRS Total dose/dose per fraction/n fractions					
27 Gy/9 Gy/3	51				50
32 Gy/8 Gy/4	51				50

**Table 2:** Gross target volume (GTV) and planning target volume (PTV) of the entire cohort of patients treated for single large brain metastases (BMs)

Median GTV and range for the entire cohort		16.3 cm^3^(3.9–64.5 cm^3^)	
GTV cm^3^	(range cm^3^)	n. patients	%
≤4	(3.9–4)	9	8.8
4.1–10	(4.36–9.76)	27	26.5
10.1–20	(10.88–19.81)	30	29.4
21–40	(22.63–39.37)	21	20.6
>40	(42.61–64.5)	15	14.7
Median PTV and range for the entire cohort		33.7 cm^3^(9.2–122.3 cm^3^)	
PTV cm^3^	(range cm^3^)	n. patients	%
<10	(9.2–10)	9	8.8
>10–24	(10.1–23.1)	27	26.5
>20–38	(21.2–36.7)	30	29.4
40–85	(40–72.37)	21	20.6
>85	(88.6–122.3)	15	14.7

### Clinical outcomes

**Fig. 2:**
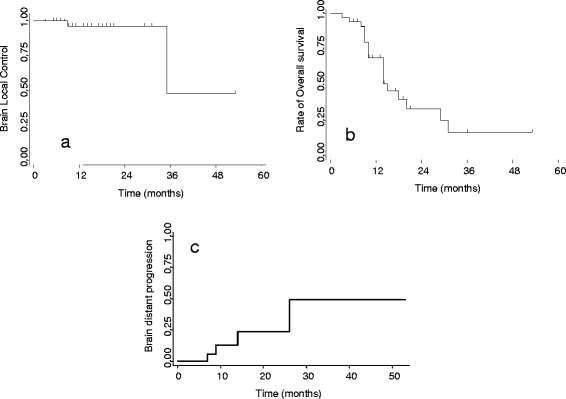
Actuarial curves for the entire cohort of patients. **a** Local control (LC) of patients treated with hypo-fractionated stereotactic radiotherapy (HSRT); **b** Brain distant progression (BDP); **c** Overall Survival (OS)

**Fig. 3:**
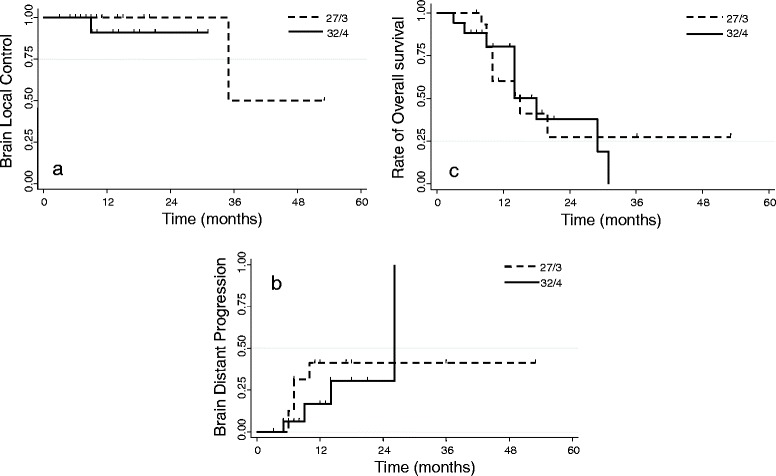
Analysis stratified according to the two dose fractionation schedules. **a** Local control (LC) of patients treated with hypo-fractionated stereotactic radiotherapy (HSRT); **b** Brain distant progression (BDP); **c** Overall Survival (OS)

With a median follow-up of 14 months (range 3–53 months), local progression at the site of HSRT occurred in 6 (5.8 %) patients at a mean time of 22 months (range 9–35 months); the total dose delivered was 27 Gy in three fractions in three patients and 32 Gy in four fractions in the others three. For the entire cohort, the median, 1 and 2-year local control rate of alive patients was 30 months, 96 and 96 %, respectively (Fig. 2a). Thirty (29 %) patients had new brain metastases at distant brain sites, and in 12 (40 %) patients progressive extracranial disease was present too. The median, 1 and 2-year rate of developing new brain metastases was 24 months (range 3–53 months), 12 and 24 %, respectively as shown in Fig. 2b. On univariate and multivariate analysis neither gender, age, KPS, histology of primary tumor, extracranial disease status (controlled/uncontrolled) at the time of HSRT, recursive partitioning analysis (RPA) class, diagnosis-specific Graded Prognostic Assessment (DS-GPA), nor size of BMs were predictive of LC or BDP. The median, 1 and 2-year OS was 14 months, 69, 33 %, respectively as shown in Fig. 2c. In relation to RPA class the median, 1 and 2-year OS was 18 months, 66.6 and 33.3 % for patients in RPA class I, 15 months, 72 and 36 % for RPA class II and 9 months, 37.5 and 0 % for RPA class III. Considering GPA, patients with score 3.5–4 had a better outcome compared with score 0–1 with a median OS of 14 months (range 3–14 months) *vs* 9 months (range 6–10 months) without statistical relevance (*p* = 0.07). The results for the analysis stratified according to the dose fractionation schedule are shown in Fig. 3. The median, 1 and 2 years LC rate for alive patients were 30 months, 100 and 100 % for 27 Gy/3 fractions *vs* (median not reached), 91 and 91 % for 32 Gy/4frs (*p* = 0.25); the median, 1 and 2 years BDP rate were (median not reached), 10 and 10 % for 27 Gy/3 fractions vs 26 months, 14.3 and 35.7 % (*p* = 0.23); the median, 1 and 2 years OS were 14 months, 60 and 27.4 % for 27 Gy/3 fractions vs 14 months, 80 and 37 % (*p* = 0.84). At the last observation time 45 (44 %) patients were alive and 57 (56 %) dead. Among dead patients 42 (73.7 %) died for progressive extracranial disease at a median time of 14 months (range 8–31 months), and in 15 (26 %) of these also brain progression occurred; 9 (15.8 %) patients died for intracranial progression at a mean time of 12 months (range 9–17 months) and 6 (10.5 %) of cerebral bleeding within 5 months from HSRT. No relations were recorded for the events in relation to the total dose delivered, and fractionation used. In fact, among nine patients died for intracranial progression, four received 27 Gy in three fractions and five patients were treated with 32 Gy in four fractions; similarly for cerebral blending, three patients received 27 Gy and three received 32 Gy. On univariate and multivariate analysis KPS and controlled extracranial disease were associated with the better prognosis (*p* <0.01).

### Toxicity

Steroid dependency occurred in 12 patients who received high-dose dexamethasone for more than 6 months in relation to the increased perilesional edema. No close correlation was recorded in respect to the RT scheme used, as half of these have received 27 Gy in three fractions and the other 32 Gy in four fractions. Among these, six patients had progressive symptoms uncontrolled by medical drugs and in such patients surgical resection was required for grade 3 radio-necrosis. No severe grade IV toxicities occurred. Minor disorders were represented by grade I-II headache in 12 patients, grade I-II hydrocephalus in six, and grade I-II ischemia cerebrovascular in six. No visual or new motor sensory deficits were recorded for patients treated for lesions in close proximity of optical nerves, chiasmas or brainstem. Six (5.8 %) cases of brain radio-necrosis occurred, and surgical resection was performed. Histological data confirmed the presence of extensive radio-necrosis that occurred at a mean time of 11 months (range 10–12 months) from HSRT. Considering the few cases observed, it was not possible to evaluate the relation between dose level and volume. In these patients the BMs were larger than 4.1 cm in maximum diameter, the PTV was between 72.4 and 122.3 cm^3^ and the total dose given was 32 Gy/4 fractions.

### Salvage treatment for intracranial/local progression

Among 36 brain relapse patients, six had local progression in site of HSRT and 30 in other brain site. The six patients with local progression had also diffuse extracranial progression and died early. About 30 patients with BDP, 21 received single section stereotactic radiosurgery (SRS) to other brain site and nine did not undergo further treatment for widespread disease progression. No patients had WBRT. Among the re-treated patients, 12 were alive at 6 months and nine patients dead within 7 months.

## Discussion

The treatment of patients with single, large brain metastases, unsuitable for surgical resection, is a challenge. Several radiation strategies have been used and described in literature: whole brain radiation therapy (WBRT), single dose stereotactic radiosurgery (SRS) and more recently multi-fraction or hypo-fractionated stereotactic radiotherapy (HSRT). WBRT has been the mainstay of treatment, but local control of single, large brain metastases is suboptimal [3, 4]. Nieder analyzed the efficacy of WBRT in 108 patients treated for 336 BMs. The LC rate was 52 % for metastases <0.5 cm^3^ and 0 % for those >10 cm^3^. Authors concluded that considering the low LC using WBRT to a total dose of 30Gy even for small metastases, patients should be treated with locally more effective dose and fractionation schedules when local control is the aim [3]. As well as achieve an inadequate LC, WBRT has several drawbacks: takes more time to deliver (2–3 weeks), thereby delaying systemic therapy that in metastatic disease is fundamental; results in loss of hair which can impact on patients’ quality of life; requires the use of steroid for a longer time, generating in some cases, many different comorbidities. In addition, the neurocognitive effects of WBRT are becoming increasingly important as improved systemic therapies increase life expectancy for patients with brain metastases. The RTOG 9508 randomized trial [4] evaluated patients with one to three newly diagnosed brain metastases receiving SRS with WBRT or SRS alone showed a decline in learning and memory function for patients who underwent WBRT compared to SRS alone (54 % vs 24 %). Although, these data revised using a different neurocognitive test showed a minor decline in neurocognitive function, this report increased the interest in omitting WBRT when possible [29]. Another RT strategy is represented by SRS, whether combined or not with WBRT that it is becoming the major treatment used for patients with solitary or limited BM (up to four). The RTOG protocol 90-05, suggested three dose levesl based on maximal tumor diameter [7], 24 Gy for lesions with maximal diameter ≤20 mm, 18 Gy in case of lesions 21–30 mm, and 15 Gy for 31–40 mm in maximum diameter. As reported by Vogelbaum a dose of 24 Gy to the tumor margin had a significantly lower risk of local failure than 15 or 18 Gy (*p* =0.0005; hazard ratio 0.277, confidence interval [CI] 0.134–0.573). With a 1-year local control rate of 85 % (95 % CI 78–92 %) compared with 49 % (CI 30–68 %) for tumors treated with 18 Gy and 45 % (CI 23–67 %) for tumors treated with 15 Gy. [8]. Chang [30] identified a 1-cm cutoff for radiosurgical control of BMs; instead using 20 Gy or more in single fraction radiosurgery, the 1- and 2-year actuarial local control rates for lesions of 1 cm (0.5 cm^3^) or less were 86 and 78 %, respectively, compared to 56 and 24 %, for lesions larger than 1 cm (0.5 cm^3^) (*p* <0.001). Other reports, defined a total tumor volume cutoff value of ≥2 cm^3^ (*p* = 0.008) as a stronger predictor of overall survival, distant brain failure and local control rate (*p* <0.001) [10–12]. Although, comparative studies of SRS and multi-fractions radiosurgery are not available, the published data confirmed that for large BMs, using a single dose radiosurgery, local control has proven to be inadequate.

These unsatisfying outcomes make HSRT a valid alternative for large brain metastases. Generally, the advantages of HSRT seem to be the following: i) the possibility to treat large brain lesions (≥2.1 cm) with lower risk of important radiation-induced neurotoxicity compared to single dose SRS; ii) the feasibility to treat lesions near to critical structures using few fractions of radiotherapy; iii) a theoretical advantage due to the re-oxygenation of hypoxic tumor cells between fractions; iv) an inferior risk of brain radio-necrosis compared to SRS; v) keeping a short treatment time compared to WBRT. In the recent years, several published papers about this issue, reported encouraging results with a 1-year LC ranging from 56 to 93 %, a median OS between 8 and 16 months and a 1-year rate of 34–66 %, and severe toxicity between 2 and 10 % as shown in table 3 [14–25]. Unfortunately, these series are extremely heterogeneous for total dose and schedules used, number of fractions, different methodology utilized, different results recorded, more than 1 BMs treated at the same time, inclusion of small and large lesions without results divided in relation to volume of BMs, and to date a suggestion on the optimal treatment is not provided. Our series includes only patients with single, large (≥2.1 cm) BM unsuitable for surgical resection, underwent HSRT. Two different schedules were used in relation to the tumor size, 27Gy in three consecutive daily fractions or 32Gy in four consecutive daily fractions. The optimal fractionation schedule for HSRT, has not yet been established. Different total doses and schedules have been used with the aim to keep a biological equivalent dose (BED) of about 60Gy_10_ (BED in Gy with α/β = 10) for tumor effects and BED of about 150Gy_3_ (BED in Gy with α/β = 3) for late effects. We chose to treat patients with a total dose corresponding to a BED_10_ greater than 50Gy considering the large volume treated. Using this approach, a local control rate of 96 % at 2 years was obtained. No differences were recorded in relation to the size of BM, 2.1–3 cm or 3.1–5 cm. Survival rates were also encouraging, with a median, 1 and 2-year OS of 14 months, 69 % and 33 %, respectively. Similar results were showed in the study of Minniti [23]. Two different schedules of HSRS for 171 BMs treated were used in relation to the tumor size. Patients with tumor <2 cm received 36Gy in three fractions while patients with tumor ≥2 cm were treated with 27Gy in three fractions. The 1-year LC was 88 % and it was similar in both groups, and the median and 1 year OS was 14.8 months and 57 % respectively. The limitation of this study was that small brain lesions, potentially suitable for SRS, were included in this analysis. The study of Fahrig is one of the largest studies about this issue [16] evaluating the outcome of 150 patients treated for 228 BM; different doses and schedules were used in relation to the size and location of the lesions. The 1-year LC was 93 % and the median and 1 year OS was 16 months and 66 % respectively but for BMs larger than 15 cm^3^ (maximum diameter >3 cm) a longer schedule of HSRT (10 fractions) was also used. The other published studies are limited for number of patients enrolled, and/or for median local control and survival not reported, and/or for inclusion of patients underwent surgical resection too [14, 15, 17, 19, 20, 24, 25]. Concerning toxicity, in different series, no severe grade III-IV neurological deficit were showed and symptomatic radio-necrosis occurred in less than 10 % of patients. Limits are that different methods were utilized for defining radio-necrosis and few studies showed data about this matter. Minniti showed that radio-necrosis occurred in nine patients (9 %), leading to severe neurologic complications in 5 (5 %) of them. The V_24Gy_ was the most significant predictor of radio-necrosis, with a cumulative risk of 14 % for volumes >16.8 cm^3^ and 4 % for volumes ≤16.8 cm^3^ [23]. Ernst-Stecken showed that side effects, i.e., increase in T2w-signal area, duration of steroid intake and size of new or progressive necrotic centre of metastasis, were dependent on the volume of normal brain irradiated with more than 4Gy per fraction (V4Gy) [15]. Kim, evaluating efficacy and toxicity of SRS compared to HSRT in 98 patients with BMs, showed similar LC and OS rates with a lower risk of toxicity for HSRT patients in comparison to those treated with SRS, despite the fact that HSRT was used for large lesions and lesions in adverse locations (17 % vs. 5 %, *p* = 0.05) [19]. In our series, no severe grade III–IV disorders were recorded and no visual or new motor-sensory deficit were observed for patients treated for lesions in close proximity of optical nerves, chiasmas or brainstem. Symptomatic radio-necrosis occurred in a limited number of patients (5.8 %) considering the large volume treated (91 % of lesions greater than 10 cm^3^), and they were patients with lesions larger than 4.1 cm in maximum diameter and volume greater than 70 cm^3^ suggesting that in these lesions a dose reduction should be considered. We are aware that our analyses has the limit of a retrospective study including patients with different histological subtypes, above all in RPA class II and in which GPA score was not represented in the entire cohort of patients. However, HSRT for patients with large brain metastases unsuitable for surgical resection has proven to be a safe and effective treatment with a high rate of local control and negligible toxicity. The use of new advanced RT techniques as volumetric modulated arc therapy permitted a high conformity for the tumor with maximum sparing of normal structures. This can be considered the main reason of the good treatment tolerance, and the lower incidence of radio-necrosis. The low toxicity recorded allowed, in case of brain distant progression, to perform a new outpatient radio-surgical treatment. More than 50 % of patients retreated are alive at about 6 months. One may argue that in our series, no WBRT was performed. Our choice, however, has proved to be winning, in fact only 12 and 24 % of patients had a BDP at 1 and 2 years, respectively. Finally, the observed OS was principally correlated with patients KPS, and controlled extracranial disease. The control of large brain metastases, however, in addition to improving quality of life, might affect survival in a selected group of patients with good KPS, controlled extracranial disease, and limited BMs.

**Table 3:** Some of larger published papers about hypo-fractionated stereotactic radiotherapy (HSRT) alone for large brain metastases

Authors	N. pts	Total dose/n.frs	mLC mos	1 yr ^b^ LC%	mOS mos	1 yr ^b^ OS%	Toxicity %
Aoyama [14]	87	35 Gy/4	NR	81	8.7	39	5
Ernst-Stecken [15]	51	35 Gy/5	7	76 %	11	NR	2
Fahrig [16]	150	30–35 Gy/5	NR	NR	16	66	10
		40 Gy/10					0
		35 Gy/7					
Narayana [17]	20	30 Gy/5	NR	70	8.5	64.5	2
Kim [19]	40	36 Gy/6	NR	69	8	34	5
Ogura [20]	39	35 Gy/5	NR	86.7		64.5	2
Minniti [23]	135	36 Gy/3	NR	88	14.8	57	7
		27 Gy/3					
Rajakesari [24]	70	25 Gy/5	17	56	10.7	NR	4.3
Croker [25]^a^	61	24 Gy/3	9	69	21	60	7

*Pts* patients, *frs* fractions, *mLC* median local control, *yr* year, *mOS* median overall survival, *mos* months

^a^Including patients underwent surgery plus hypofractionated stereotactic radiosurgery (HSRS)

^b^Actuarial

## Conclusion

In conclusion, in patients with single, large BM unsuitable for surgical resection, HSRT is a safe and feasible treatment, with good brain local control and limited toxicity.

## Ethics approval and consent to participate

All procedures performed in studies involving human participants were in accordance with the ethical standards of the institutional and/or national research committee and with the 1964 Helsinki declaration and its later amendments or comparable ethical standards. This study was based on a retrospective analysis of treatment charts and received approval by the Humanitas Hospital Ethical Committee. All patients, during admission, signed a consent to the use of their data for scientific scopes.

## Consent for publication

Not applicable.

## Availability of data and materials

Datasets can be retrieved from authors upon formal request from interested readers. Datasets cannot be directly shared on public repositories due to the national personal data protection act.

## Abbreviations

4D, four-dimensional; AAPM, American association of physicists in medicine; BDP, brain distant progression; BED, biological equivalent dose; BM, brain metastases; CI, confidence interval; CTV, clinical target volume; DS_GPA, diagnosis-specific graded prognostic assessment; GTV, gross tumor volume; HSRT, hypo-fractionated stereotactic radiotherapy; ICRU, international commission of radiological units; KPS, Karnofsky performance scale; LC, local control; MRI, magnetic resonance imaging; OAR, organs at risk; OS, overall survival; PTV, planning target volume; RPA, recursive partitioning analysis; RT, radiotherapy; RTOG, radiation therapy oncology group; SBRT, stereotactic body radiation therapy; SCLC, small cell lung cancer; SRS, stereotactic radiosurgery; TPS, treatment planning system; WBRT, whole brain radiation therapy
